# A Review of the Role of Stereotactic Radiosurgery and Immunotherapy in the Management of Primary Central Nervous System Tumors

**DOI:** 10.3390/biomedicines10112977

**Published:** 2022-11-19

**Authors:** Eric J. Lehrer, Brianna M. Jones, Kunal K. Sindhu, Daniel R. Dickstein, Mira Cohen, Stanislav Lazarev, Alfredo Quiñones-Hinojosa, Sheryl Green, Daniel M. Trifiletti

**Affiliations:** 1 Department of Radiation Oncology, Icahn School of Medicine at Mount Sinai, New York, NY 10029, USA; 2 Department of Neurosurgery, Mayo Clinic, Jacksonville, FL 32224, USA; 3 Department of Radiation Oncology, Mayo Clinic, Jacksonville, FL 32224, USA

**Keywords:** stereotactic radiosurgery, immunotherapy, neurosurgery, radiation oncology, glioma, meningioma, glioblastoma

## Abstract

Stereotactic radiosurgery (SRS) and immune checkpoint inhibitors (ICIs) are widely used in the management of brain metastases. These therapies are commonly administered concurrently; as SRS may enhance anti-tumor immunity and responsiveness to ICIs. However, the use of ICIs with and without SRS in the management of primary brain tumors remains a controversial topic. Meningiomas are the most common nonmalignant and extra-parenchymal brain tumor, which often respond well to surgery and radiotherapy. However, higher grade meningiomas tend to be resistant to these treatments, and the use of chemotherapy and targeted agents in this setting have yielded disappointing results. Thus, there is heightened interest in the utilization of ICIs. Glioblastoma is the most common malignant primary intraparenchymal brain tumor. It is associated with a grim prognosis with a median overall survival of approximately 20 months, despite optimal therapy. While SRS in the adjuvant setting, and ICI in the recurrent setting, have failed to demonstrate a survival benefit, SRS in the preoperative setting has the potential to enhance anti-tumor immunity and responsiveness to ICIs. Thus, these treatments represent an attractive option to add to the armamentarium of meningioma and glioblastoma management. In this review, we provide a detailed overview of the evidence supporting the use of ICIs and SRS in each of these settings.

## 1. Introduction

Over the past decade, there have been significant advances in systemic cancer therapies. One of the most notable of these advancements are immune checkpoint inhibitors (ICIs), which have resulted in improved overall survival (OS) in multiple advanced malignancies [1,2,3]. Additionally, multiple studies have suggested that ablative doses of radiotherapy (e.g., stereotactic radiosurgery [SRS]) are able to enhance the efficacy of ICIs by augmenting anti-tumor immunity [4,5,6,7,8,9,10,11]. Thus, there has been great interest in combining these two therapies across different primary tumor types. However, to date, the most widely studied and common intracranial application is in the management of brain metastases [4,5,6,7,8,9,12].

Meningiomas are the most common benign primary central nervous system (CNS) tumor [13]. Stereotactic radiosurgery and conventionally fractionated radiation therapy (RT) have demonstrated excellent rates of local control (LC) and progression-free survival (PFS) in low- and intermediate-risk meningiomas following surgical resection [14,15,16]. However, high-risk lesions have 3-year PFS rates as low as 30% following RT [17]. The use of chemotherapeutic and targeted agents in this setting has had minimal success [18,19]. Thus, novel approaches are needed to increase the therapeutic ratio. Due to their location outside of the blood–brain barrier (BBB), which permits exposure to peripheral immune cells, ICIs are an attractive treatment option for higher grade meningiomas that have been refractory to traditional therapies [20,21,22].

Glioblastoma is the most common primary malignant brain tumor, and is associated with a very poor prognosis, with a median OS of 15–21 months despite optimal therapy [13]. While SRS has been explored in the adjuvant setting and yielded disappointing results [23], there has been recent interest in incorporating preoperative SRS as a dose-escalation strategy [24]. Furthermore, preclinical studies and case reports have suggested that ICI therapy may enhance anti-tumor immune responses against glioblastoma, which can potentially lead to improved outcomes [25,26,27,28,29,30]. However, to date, clinical trials utilizing ICIs have been disappointing [31,32,33].

In the forthcoming sections, we provide a detailed overview of preclinical and clinical data, as well as ongoing trials utilizing SRS and ICIs in the management of the most common brain tumors in adults, meningiomas and glioblastoma.

## 2. Meningioma

Meningiomas are the most common primary benign CNS tumor [13]. According to the 2021 World Health Organization (WHO) criteria, meningiomas are classified into 15 distinct variants based on histologic and molecular features, with a grading system applied regardless of subtype [34]. Grade 1 is defined as having mitoses <4 per 10 high-power fields (hpf), grade 2 is defined as having ≥ 4–19 mitoses per 10 hpf, and grade 3 is defined as >20 mitoses per 10 hpf. Tumor grade is predictive of recurrence and progression [14,15,17,34,35]. Patients with grade 1 meningiomas comprise the majority of tumors, with a 10-year OS of 80–90% and PFS of 75–90% [13,35,36]. Approximately 15–18% of meningiomas are WHO grade 2 (atypical), and have a 10-year survival of 53–79% and a PFS of 23–78% [1,3,5]. WHO grade 3 (anaplastic/malignant) meningiomas comprise 1–3% of all tumors, and have a 10-year OS of 14–34% and a PFS of 0–10% [35,36]. 

There have been efforts to incorporate molecular and genomic classification into diagnosis in order to better identify targeted therapeutic agents [37,38]. For instance, the TERT promoter activating mutation and CDKN2A/B deletion is found in anaplastic, CNS WHO grade 3 tumors, and is associated with poorer survival [37,39]. The use of CDK4/6 inhibitors plus radiation for high grade meningiomas with CDKN2A/B homozygous gene deletion has demonstrated encouraging results in pre-clinical murine models [40]. There are several other genetic alterations commonly associated with meningiomas, including: NF2, AKT1, TRAF7, SMO, PIK3CA; KLF4, SMARCE1, and BAP1 in subtypes; H3K27me3 [34]. A recent study demonstrated higher PD-L1 levels in tumors with specific genetic mutations (i.e., TRAF7 mutations) [41]. Tumor mutational burden has been previously demonstrated to be associated with higher presence of neoantigens, eliciting a strong anti-tumor immune response and, therefore, a response to ICIs [42,43]. Beyond genetic mutations, data suggest that epigenetic changes contribute to disease development and progression in meningiomas. A recent retrospective analysis proposed a DNA methylation based-classification system, which was more homogenous and predictive of recurrence and prognosis when compared to the WHO classification system [37]. Nassiri et al. proposed four molecular groups as well as the downstream gene expression pathways (MG1: immunogenic, MG2: benign NF2 wild-type, MG3: hypermetabolic, and MG4: proliferative) that guided therapeutic options. For instance, vorinostat selectively decreased cell survival in MG4 tumors, but not in the other molecular groups [38]. Future studies will likely incorporate these molecular features into diagnosis in order to help guide treatment.

## 3. Meningioma Treatment

The mainstay of treatment for meningiomas is maximal surgical resection, with the extent of resection being predictive of recurrence [44]. Definitive or adjuvant RT, including conventionally fractionated external beam radiotherapy (EBRT), SRS, and hypofractionated SRS, are used to improve outcomes in select settings where tumors are not amenable to surgery, those that are subtotally resected, and in those that recur following resection [15,16,17,45]. On the Radiation Therapy Oncology Group (RTOG) 0539 trial, patients were stratified into low, intermediate, and high-risk groups, and subsequently received risk-adapted postoperative EBRT [14,15,17]. Adjuvant radiation was well-tolerated, and demonstrated superior LC and PFS in intermediate (recurrent grade 1 or new WHO grade 2 with gross total resection [GTR] or subtotal resection [STR]), as well as in high risk (any WHO grade 3, recurrent grade 2, new grade 2 with STR) cohorts, compared to historical controls [15,17]. Stereotactic radiosurgery has also been used to treat small, low-grade tumors (<10 cm^3^ in volume or <3 cm in diameter) with distinct margins that are at a sufficient distance from important functional areas, with tumor control rates exceeding 90% [16]. For tumors that are close to critical structures (e.g., optic apparatus, brainstem), hypofractionated SRS has been used with favorable results [45]. The role of radiosurgery for WHO grade 2/3 and recurrent tumors is more controversial due to the suboptimal tumor control rates in some series. and the need to treat the entire surgical bed in addition to gross disease, which is not often feasible with SRS [46]. However, several retrospective studies and single institution studies have demonstrated acceptable LC with margin doses of 12–20 Gy [46,47,48,49]. Additionally, there have been efforts to identify optimal subgroups of patients with higher grade tumors that may benefit from SRS [50]. While meningioma is generally considered a benign disease, there are certain subsets of tumors that appear to be more aggressive. Recurrent or incompletely resected tumors are difficult to treat, with chemotherapy having a limited role [18]. There is emerging evidence that incorporation of ICIs and radiation may improve outcomes in certain aggressive subtypes of meningioma [20].

### 3.1. Immune Checkpoint Inhibitors

There is evidence of decreased anti-tumor immune response in meningiomas. Several immune cell populations have been identified in meningiomas, such as macrophages, microglia, and lymphocytes, with most tumor-infiltrating immune cells being antigen-experienced [21,51,52,53]. There is also expression of inhibitory ligands PD-L1/PD-L2 and CTLA-4, contributing to T-cell exhaustion, and proportional to tumor grade [53]. One study found that PD-L1 expression was an independent predictor of a worse OS [54]. Meningiomas also arise from arachnoid cap cells of the inner dura layer outside the BBB, thus making them readily accessible to peripheral immune cells. Further, meningiomas often involve, or are in close proximity to, venous sinuses. Given the recently discovered intracranial lymphatic system that abuts the dural sinuses, meningiomas in close proximity to venous sinuses may have access to a lymphatic system and, thus, may interact with peripheral antigens [55]. Taken together, these findings suggest that ICIs may be useful in the management of higher grade meningiomas. 

The blockade of inhibitory ligands and receptors with ICIs has demonstrated durable antitumor effects and disease control in several tumor types [56,57,58]. The role of systemic therapy for meningiomas is controversial and limited to recurrent or progressive disease not amenable to surgery or radiation. Several studies have examined the role of chemotherapy, targeted agents, angiogenesis inhibitors, and hormone therapy with no or minimal improvement in outcomes [18]. In contrast, ICIs have demonstrated favorable outcomes in early studies. Preliminary results from the single-arm open-label phase II trial (NCT03279692), evaluating the efficacy of pembrolizumab in 25 patients with recurrent and progressive grade 2/3 meningiomas, met the primary endpoint, achieving a PFS-6 rate of 0.48 and median PFS of 7.6 months [59]. In another retrospective analysis of eight patients with recurrent meningioma that received anti-PD-1 therapy, there was an increased PFS and OS in patients with WHO grade 3 and expression of PD-1/PD-L1 [60]. Several other ongoing trials (NCT02648997, NCT03173950) will evaluate the role of ICIs for surgery and radiation-refractory meningiomas. 

### 3.2. Combining Stereotactic Radiosurgery and Immune Checkpoint Inhibitors

The addition of SRS to ICIs has been used synergistically for other primary and metastatic CNS tumors [4,5,6,7,8,9,12,24]. Preclinical data suggest that radiation-induced immunogenic cell death increases antigen presentation and activation of immune cells and, in combination with ICIs, subverts the immunosuppressive tumor microenvironment [61,62]. Based on previous evidence of enhanced anti-tumor immune response with this combination therapy in CNS tumors, the use of radiation and ICIs for meningiomas is actively being investigated in several ongoing trials, as listed in Table 1. 

## 4. Glioblastoma

Glioblastoma is the most common malignant primary brain tumor [13,63]. These tumors are extremely aggressive and are highly resistant to treatment, with the median OS ranging from 15 to 21 months [64,65,66,67,68,69,70]. The classification of glioblastoma has recently been updated. In the 2021 WHO update, isocitrate dehydrogenase (IDH)-mutant glioblastoma are now classified as *IDH-mutant astrocytomas* [34]. Additionally, the 2021 update further incorporates molecular markers and is no longer fully histological, as the presence of a *CDKN2A/B* homozygous deletion automatically results in a WHO grade 4 diagnosis. Finally, in the setting of *IDH-wildtype diffuse astrocytic gliomas*, the presence of any of the following: *TERT* promotor mutation, *EGFR* gene amplification, and a combined chromosomal *+7/-10* alternation, is sufficient to assign the diagnosis of *glioblastoma, IDH-wildtype*. While it does not factor into tumor classification, methylation of the O^6^-methylguanine-DNA methyltransferase (MGMT) gene promotor is a favorable prognostic factor in patients with glioblastoma [71]. MGMT is a DNA repair enzyme that antagonizes the effects of alkylating agents, such as temozolomide (TMZ) and carmustine (BCNU). The presence of MGMT promotor methylation has demonstrated a roughly 6-month OS benefit in modern studies, where patients were treated with optimal therapy [66,67,71]. 

## 5. Glioblastoma Treatment

The mainstay of treatment for glioblastoma involves a multimodality approach consisting of maximal surgical resection, followed by adjuvant RT with concurrent TMZ, and then adjuvant TMZ and tumor-treating fields (TTF) [65,66,67,68,69]. While this multimodality regimen is associated with the best outcomes, multiple factors must be taken into account when selecting an ideal treatment regimen, such as patient performance status, prognosis, and age. Thus, de-intensified strategies are commonly employed in the elderly and in patients with poor performance status [72,73,74,75].

Maximal tumor resection is the fundamental component of glioblastoma treatment, as the extent of resection has been shown to be a strong predictor of OS [76,77,78,79,80]. In 2005, Stupp et al. published the results of a phase 3 trial, where patients with glioblastoma following resection were randomized to: (1) RT alone to 60 Gy in 30 fractions, or (2) RT to 60 Gy in 30 fractions delivered with concurrent TMZ, followed by adjuvant TMZ [67]. A significant improvement in median OS was observed, favoring the TMZ arm (12.1 months versus 14.6 months). Thus, this treatment paradigm is now considered to be the standard of care in appropriately selected patients with glioblastoma. Additionally, the incorporation of TTF into the treatment paradigm for glioblastoma has demonstrated an improvement in OS from 16 months to 20.9 months [68,69]. However, the use of TTF is a controversial topic in the neuro-oncology community, and its incorporation into treatment plans varies widely [81]. 

### 5.1. Immune Checkpoint Inhibitors

Several preclinical studies have explored the role of ICIs in experimental glioma models with promising results [25,26,27,28,29,82]. In 2007, Fecci et al. published a study utilizing a mouse glioma model, where anti-CTLA-4 therapy conferred an 80% long-term survival in mice and reduced the population of infiltrating immunosuppressive regulatory T-cells [28,29]. Another preclinical study utilized a glioblastoma stem cell mouse model, where an IL-12 expressing oncolytic virus, anti-PD-1, and anti-CTLA-4 therapy were administered [30]. A cure rate of 89% was observed, and 100% of those cured remained alive at 96 days following tumor re-challenge, suggesting both a survival advantage with this therapy and immunological memory.

Unfortunately, clinical trials utilizing ICIs in glioblastoma have yielded disappointing results. Checkmate 143 was a phase 3 trial that randomized patients with recurrent glioblastoma who received standard treatment with RT and TMZ to (1) nivolumab or (2) bevacizumab [32]. With a median follow-up of 9.5 months, the median OS was 9.8 versus 10.0 months for the nivolumab and bevacizumab groups, respectively. Additionally, treatment-related adverse events were similar between the study arms. Another randomized study comparing pembrolizumab alone to pembrolizumab plus bevacizumab in the setting of recurrent glioblastoma was published in 2021 [33]. No significant differences in median OS were observed, suggesting that pembrolizumab monotherapy or in addition to bevacizumab was ineffective in the setting of recurrent glioblastoma. In 2021, the recurrent glioblastoma subgroup analysis of the KEYNOTE-028 trial was published [83]. Twenty-six patients were enrolled and received pembrolizumab 10 mg/kg every two weeks for up to two years. An overall response rate of 8% was observed, with a median PFS of 2.8 months and a median OS of 13.1 months. These findings suggest that pembrolizumab monotherapy exhibited anti-tumor activity in a small subset of patients with recurrent glioblastoma.

There are multiple reasons for the ineffectiveness of ICI therapy in the setting of glioblastoma. First, glioblastomas have few T-cells and contain large populations of immunosuppressive tumor-associated macrophages, which are characteristic of IDH-wildtype tumors [84,85]. Second, high concentrations of immunosuppressive myeloid cells are characteristic of glioblastoma [86]. Third, high tumor mutational load is rarely present in glioblastoma. Taken together, these characteristics provide insight into the immunologically “cold” nature of glioblastoma [42,43,87].

In a 2019 study by Cloughesy et al., 35 patients with recurrent surgically resectable glioblastoma were randomized to neoadjuvant pembrolizumab with continued adjuvant therapy or adjuvant pembrolizumab alone [88]. The median OS was 228.5 versus. 417 days, favoring the neoadjuvant arm, *p* = 0.04. Additionally, neoadjuvant pembrolizumab was associated with upregulation of local and systemic antitumor immune responses when compared to adjuvant treatment alone. Thus, this may represent a novel treatment paradigm in the management of recurrent GBM. Neoadjuvant ICI therapy is presently being explored in the upfront setting in a phase 2 clinical trial at the Mayo Clinic (NCT03197506). Patients will either receive a single cycle of neoadjuvant pembrolizumab, followed by concurrent and adjuvant pembrolizumab in combination with TMZ and RT, or concurrent and adjuvant pembrolizumab in combination with TMZ and RT.

### 5.2. Adjuvant Stereotactic Radiosurgery

Given the poor prognosis of glioblastoma and its tendency to recur within two centimeters of the surgical resection cavity, there has been increased interest in recent years in radiation dose escalation, an attribute that, theoretically, could be offered by SRS [89,90,91]. However, evidence for such an approach is currently lacking [24,64]. RTOG 9305 randomized 203 patients with supratentorial glioblastoma measuring 4 cm or less to receive postoperative SRS (with the dosing based on RTOG 9005) versus observation, after receiving conventionally fractionated RT to a dose of 60 Gy in 30 fractions as well as BCNU. This study found that the median OS between the two groups did not differ (13.5 versus 13.6 months in the SRS group and observation groups, respectively) [23,92]. However, caution is warranted in extrapolating these results to the modern setting, as the trial did not utilize TMZ [67]. Additionally, it should be noted that the impact of SRS sequencing in patients with glioblastoma is unclear [65].

### 5.3. Preoperative Stereotactic Radiosurgery and Synergy with Immune Checkpoint Inhibitors

Preoperative SRS, which has been studied in the setting of brain metastases, has been largely unexplored in the management of glioblastoma. This treatment paradigm may offer several advantages over postoperative SRS. First, preoperative SRS permits smaller target volumes than postoperative SRS, allowing for greater sparing of surrounding healthy tissues and a lower risk of treatment-related toxicities, such as radiation necrosis [24]. Second, the concentration of oxygen is higher in intact tissues, thus allowing for more efficient cell kill via radiation-induced double-stranded DNA breaks [93]. Third, preoperative SRS allows for analysis of irradiated tissue, potentially allowing for the examination of the post-SRS tumor microenvironment and identification of repair pathways, thereby facilitating the development of novel treatments. Lastly, preoperative SRS may entail a lower risk of leptomeningeal dissemination [94].

While preoperative SRS is an exciting treatment paradigm, there are multiple scenarios which can make this intervention difficult to administer. First, patients with glioblastoma may present with significant mass effect, requiring urgent surgical intervention and decompression. Second, glioblastoma can be several centimeters in diameter, which can make SRS challenging to administer while obeying nearby healthy tissue dose tolerances. Additionally, in patients who are receiving ICIs, the use of corticosteroids needs to be carefully considered, as their immunosuppressive nature can limit the efficacy of ICIs. Finally, performing SRS without histologic confirmation remains a controversial subject. Thus, careful patient selection is essential.

Another potential benefit of preoperative SRS in patients with glioblastoma and high-grade gliomas is a potential increase in anti-tumor immunity, which may be further enhanced by ICIs [24]. Preclinical studies have demonstrated that RT can induce immunogenic effects. Specifically, RT can act as an anti-tumor vaccine by increasing the release of tumor-related antigens, thereby allowing for the priming of CD8^+^ T-cells and facilitating the development of an adaptive immune response [95]. In a 1994 study by Klein et al., where escalating radiation doses were delivered to glioblastoma specimens, exposure to RT was linked to an increase in major histocompatibility complex class I antigen expression, suggesting an increase in cytotoxic T-cell activity against glioblastoma [96]. Additionally, a recent study by Soltani et al. suggested that glioblastomas and low grade meningiomas share similarities in their tumor microenvironments which are associated with immunosuppression [97].

Ablative doses of RT have also been shown to increase antigen presentation and activation of CD8^+^ T-cells through various mechanisms [4,5,95,98,99,100,101]. Additionally, synergies may be unlocked with the addition of ICIs. A 2012 study by Zeng et al., for example, in which mouse models of glioblastoma were treated with anti-PD-1 therapy ± SRS to a dose of 10 Gy, found that mice that received both therapies experienced a near doubling of median overall survival as compared to those that received no therapy, anti-PD-1 therapy alone, or SRS alone. These findings suggest that the anti-tumor immunity induced by SRS may be further amplified with ICIs [28]. Thus, combining SRS, particularly in the preoperative setting, with ICIs in the treatment of patients with glioblastoma and high-grade gliomas may prove to be an attractive therapeutic combination, with the potential to improve patient outcomes. The NeoGlioma trial (NCT05030298) is a phase I/IIA study, taking place at Mayo Clinic Florida, which is presently investigating the role of preoperative SRS in the management of glioblastoma and high-grade glioma.

## 6. Conclusions

Higher grade meningiomas and glioblastomas remain difficult to treat, and are often refractory to existing standard of care therapies. The incorporation of ICIs into the treatment paradigm of meningiomas and glioblastoma, and preoperative SRS in glioblastoma have demonstrated promising results in largely preclinical settings, and remain an ongoing area of clinical investigation. 

## Figures and Tables

**Table 1:** Ongoing clinical trials combining radiation with ICIsin meningioma.

Study Title	Study Site	Trial Registration Number	Treatment Arms	Treatment Details	Status
A Phase II Study of Stereotactic Radiosurgery in Conjunction With the PD-1 Inhibitor, Pembrolizumab for the Treatment of Recurrent Meningioma	UCSF	NCT04659811	SRS +/− Pembrolizumab	SRS: Dose/fractionation based on size </=8cc: Margin dose of 15–20Gy >8cc-20cc: Total dose 25–30Gy/5 fx Pembrolizumab: 200mg give IV on day 1 (to -1) then every 3 weeks	Recruiting
A Phase I/II Study of Nivolumab Plus or Minus Ipilimumab in Combination with Multi-Fraction Stereotactic Radiosurgery for Recurrent High-Grade Radiation-Relapsed Meningioma	Multi-institution	NCT03604978	SRT + Nivolumab +/− Ipilimumab	Nivolumab: 480 mg IV every 4 weeks for 6 months or 3mg/kg IV every 2 weeks Ipilimumab 1mg/kg IV every 6 weeks x 4 Multi-fraction SRS: 8Gy/3fx QOD	Recruiting
A Phase Ib Study of Neoadjuvant Avelumab and Hypofractionated Proton Radiation Therapy Followed by Surgery for Recurrent Radiation-refractory Meningioma	Washington University School of Medicine	NCT03267836	Neoadjuvant Avelumab and Hypofractionated proton therapy followed by surgery	Avelumab: 10mg/kg every 2 weeks for 3 months Proton RT: 20CGE/5 fractions	Active, not recruiting

Abbreviations: IV: Intravenous, QOD: every other day, Gy: gray, fx: fraction, CGE: cobalt gray equivalent, 3D-CRT: three-dimensional conformal radiation therapy, ICIs: immune checkpoint inhibitors, IMRT: intensity modulated radiation therapy, EBRT: external beam radiation therapy, RT: radiation therapy.

## Data Availability

Not applicable.
